# Possible links between the lag structure in visual cortex and visual streams using fMRI

**DOI:** 10.1038/s41598-019-40728-x

**Published:** 2019-03-12

**Authors:** Bo-yong Park, Won Mok Shim, Oliver James, Hyunjin Park

**Affiliations:** 1 Department of Electrical and Computer Engineering, Sungkyunkwan University, Suwon, 16419 Korea; 2 Center for Neuroscience Imaging Research, Institute for Basic Science (IBS), Suwon, 16419 Korea; 3 Department of Biomedical Engineering, Sungkyunkwan University, Suwon, 16419 Korea; 4 School of Electronic and Electrical Engineering, Sungkyunkwan University, Suwon, 16419 Korea

## Abstract

Conventional functional connectivity analysis using functional magnetic resonance imaging (fMRI) measures the correlation of temporally synchronized brain activities between brain regions. Lag structure analysis relaxes the synchronicity constraint of fMRI signals, and thus, this approach might be better at explaining functional connectivity. However, the sources of the lag structure in fMRI are primarily unknown. Here, we applied lag structure analysis to the human visual cortex to identify the possible sources of lag structure. A total of 1,250 fMRI data from two independent databases were considered. We explored the temporal lag patterns between the central and peripheral visual fields in early visual cortex and those in two visual pathways of dorsal and ventral streams. We also compared the lag patterns with effective connectivity obtained with dynamic causal modeling. We found that the lag structure in early visual cortex flows from the central to peripheral visual fields and the order of the lag structure flow was consistent with the order of signal flows in visual pathways. The effective connectivity computed by dynamic causal modeling exhibited similar patterns with the lag structure results. This study suggests that signal flows in visual streams are possible sources of the lag structure in human visual cortex.

## Introduction

Functional magnetic resonance imaging (fMRI) is a powerful tool that measures brain activities by detecting changes in blood-oxygen-level-dependent (BOLD) signals. Functional connectivity analysis using resting-state fMRI (rs-fMRI) is the representative method for quantifying complex brain networks using the BOLD fluctuations^1–3^. The current functional connectivity studies are based on the assumption that the brain activities are temporally synchronized^4,5^. However, previous studies found asynchronous intrinsic activities in rodent brain using voltage-sensitive dye imaging and optical imaging^6–9^. They observed that the brain activity in one region propagates to other regions with variable temporal delay^6–9^. These studies suggest that the brain activities could be modeled using the appropriate temporal lags^6–9^. Mitra *et al*. expanded the concept of temporal lag to jointly consider the lag patterns among many brain regions in human^4,5^. They computed the lag structure in fMRI signals by calculating time delayed cross correlations between the time series of every voxel^4,5^. Mitra *et al*. hypothesized that the infra-slow neural processes might cause the temporal lag of the fMRI signal, which contradicts many existing studies assuming that fMRI signal is affected by the high-frequency neural activity filtered through a hemodynamic process^5,6,10,11^. However, whether the lag structure actually reflects the neural signal is still controversial^11^.

A previous animal study found traveling waves with delay patterns in cat and monkey brains by giving invasive stimuli to their visual cortex^12^. Another study found a significant association between the propagation pattern of the neuronal calcium and BOLD signals in mouse, directly implying that BOLD signals reflect neural processes^6^. Previous animal studies demonstrated a direct relationship between BOLD and neural signals^6,12^. However, none have explored the direct links between the lag structure in fMRI signals and neural processes in the human brain. Only a few studies of lag structure that indirectly explored the temporal lag patterns for the human brain exist^4,5,13^. Thus, the possible sources of temporal lag patterns in the human brain are primarily unknown.

In this study, we applied the lag structure approach to the fMRI signals of the human visual cortex to identify the possible sources of lag structure in the human visual cortex. We chose the visual cortex as it is a well-studied region^14–19^, and will allow us to use the results from established studies to possibly explain the lag structure in the visual cortex. A previous study reported the lag patterns in the human visual cortex using magnetoencephalography (MEG) and found that the stimuli in the central visual field revealed earlier onset latencies in the primary visual cortex (V1) compared to the stimuli in peripheral visual field^20^. The results suggested that the central visual field of V1 might respond earlier to the stimuli than the peripheral visual field^20^. They also observed that the information from the early visual cortex propagated to the higher visual areas in two different visual pathways of dorsal and ventral streams indicating the temporal lags in visual cortex might be associated with visual streams^20^. Thus, we focused on identifying the factors that could explain the lag structure in the human visual cortex by comparing the lag structure between the central and peripheral visual fields in the early visual cortex and that between the two visual pathways of dorsal and ventral streams. To validate the reliability of the lag structure analysis, we compared the lag structure results with dynamic causal modeling (DCM)^21–25^. DCM is still controversial, but it is one of the few well-known approaches to estimate directional interactions (i.e., effective connectivity) between different brain regions. We hypothesized that if the order of lag structure flow reflected the order of flow in visual streams, a similar order of flow might appear in the results of effective connectivity obtained with DCM.

## Results

### Lag structure in early visual cortices

**Figure 1:**
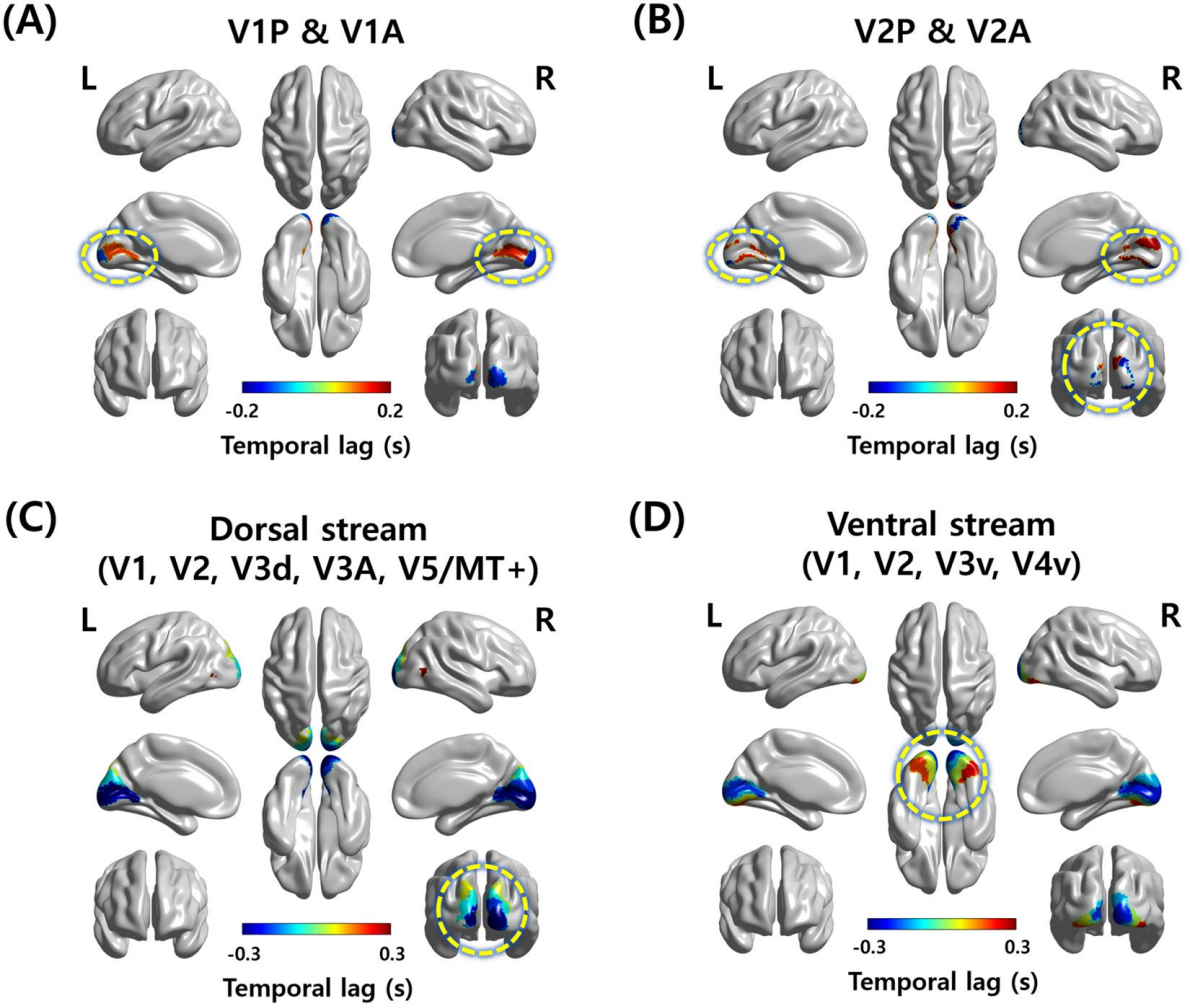
The patterns of the temporal lag among ROIs. (**A**) The visualization of the temporal lag propagation path between V1A and V1P and (**B**) V2A and V2P. (**C**) The visualization of the temporal lag propagation path of the dorsal and (**D**) ventral streams. The most visible parts are marked with yellow dotted circles. A, anterior; P, posterior; L, left hemisphere; R, right hemisphere.

In the current study, we analyzed 655 rs-fMRI data with a fast repetition time (TR) of 0.72 s from the Human Connectome Project (HCP) database^26^. We first explored the lag structure between central and peripheral visual fields in early visual cortices of V1 and secondary visual cortex (V2). The V1 and V2 were divided into the anterior and posterior subregions using the connectivity-based parcellation approach^27^. The anterior and posterior subregions were well matched with peripheral and central visual fields, respectively^27^. The temporal lags between the anterior and posterior subregions of V1 and V2 (i.e., anterior V1 [V1A], posterior V1 [V1P], anterior V2 [V2A], and posterior V2 [V2P]) were computed. As the regions of interest (ROI) sets consisted of two regions (V1A/V1P and V2A/V2P), a total of 1,310 (=2 × 655) propagation paths were possible for V1 and V2, respectively. Among the 1,310 possible paths, the path that propagated from the left V1P to V1A was observed 1,078 times (mean temporal lag = 0.2453 s), 930 times from the right V1P to V1A (mean temporal lag = 0.2707 s), 934 times from the left V2P to V2A (mean temporal lag = 0.2903 s), and 930 times from the right V2P to V2A (mean temporal lag = 0.3504 s). The results showed that the pattern of the temporal lag primarily propagated from the posterior (i.e., central visual field) to the anterior (i.e., peripheral visual field) subregion of V1 and V2 (Fig. 1A,B) consistent with the previous study^20^.

### Comparison with visual streams

**Figure 2:**
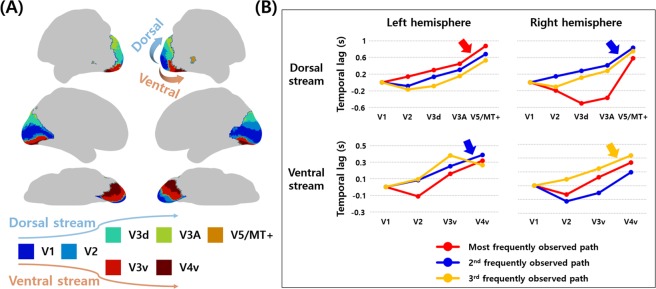
The top three frequently observed propagation paths in dorsal and ventral streams. (**A**) The ROIs of dorsal and ventral streams. (**B**) The top three frequently observed paths according to the temporal lag values are plotted with different colors. The temporal lag value in V1 was set to zero and those for other regions were moderated. The paths that were consistent with the order of the visual streams are reported with arrows.

In addition to the central and peripheral visual fields, we applied the lag structure approach to the regions of the two visual pathways and compared with the order of the signal flows in dorsal and ventral streams, respectively. The dorsal stream consisted of five ROIs of V1, V2, dorsal V3 (V3d), V3A, and V5/middle temporal complex (MT+), and thus a total of 3,275 (=5 × 655) propagation paths were possible. Similarly, 2,620 (=4 × 655) propagation paths were possible for the ventral stream (V1, V2, ventral V3 [V3v], and V4v). The most frequently observed path of the left dorsal stream was observed 284 times, and it started from V1 and propagated to V2, V3d, V3A, and V5/MT+ (Table 1 and Fig. 1C). The same path was observed 224 times with the second-highest frequency in the right hemisphere (Table 1 and Fig. 1C). In the ventral stream, the path that propagated from V1 to V2, V3v, and V4v was observed 307 times with the second-highest frequency for the left hemisphere, and 247 times with the third-highest frequency for the right hemisphere (Table 1 and Fig. 1D). The top three frequently observed propagation paths in the dorsal and ventral streams are plotted in Fig. 2. The propagation paths that were the same as the visual streams were not observed with the most frequency, but they were observed in the top three frequent cases. The results suggest that the visual streams could be possible sources of lag structure in the human visual cortex.

**Table 1:** The top three frequently observed propagation paths in dorsal and ventral streams.

Stream	Frequency	Propagation paths and temporal lag (s)				
Left dorsal	***1*** ^***st***^ ***(284 times)***	***V1***	***V2***	***V3d***	***V3A***	***V5/MT*** **+**
		**−** ***0.3806***	**−** ***0.2428***	**−** ***0.0813***	***0.0671***	***0.4945***
	2^nd^ (257 times)	V2	V1	V3d	V3A	V5/MT+
		−0.2695	−0.1833	−0.0499	0.1188	0.4980
	3^rd^ (187 times)	V2	V3d	V1	V3A	V5/MT+
		−0.2362	−0.1537	−0.0651	0.0860	0.4655
Right dorsal	1^st^ (231 times)	V3d	V3A	V2	V1	V5/MT+
		−0.3620	−0.2285	−0.0538	0.1449	0.7259
	***2*** ^***nd***^ ***(224 times)***	***V1***	***V2***	***V3d***	***V3A***	***V5/MT*** **+**
		**−** ***0.3393***	**−** ***0.1937***	**−** ***0.0642***	***0.0691***	***0.4935***
	3^rd^ (189 times)	V2	V1	V3d	V3A	V5/MT+
		−0.3268	−0.2212	−0.1057	0.0601	0.5335
Left ventral	1^st^ (381 times)	V2	V1	V3v		V4v
		−0.1972	−0.0845	0.0739		0.2317
	***2*** ^***nd***^ ***(307 times)***	***V1***	***V2***	***V3v***		***V4v***
		**−** ***0.1952***	**−** ***0.1118***	***0.0542***		***0.1901***
	3^rd^ (251 times)	V1	V2	V4v		V3v
		−0.1884	−0.0967	0.0739		0.1915
Right ventral	1^st^ (501 times)	V2	V1	V3v		V4v
		−0.2126	−0.0837	0.0397		0.2513
	2^nd^ (348 times)	V2	V3v	V1		V4v
		−0.2006	−0.0772	0.0272		0.2198
	***3*** ^***rd***^ ***(247 times)***	***V1***	***V2***	***V3v***		***V4v***
		**−** ***0.2113***	**−** ***0.1185***	***0.0373***		***0.2262***

The regions and corresponding mean temporal lag values (unit in seconds) were reported. The paths that showed the same propagation order with visual streams were reported in bold italic.

### spDCM results in early visual cortices and visual streams

**Figure 3:**
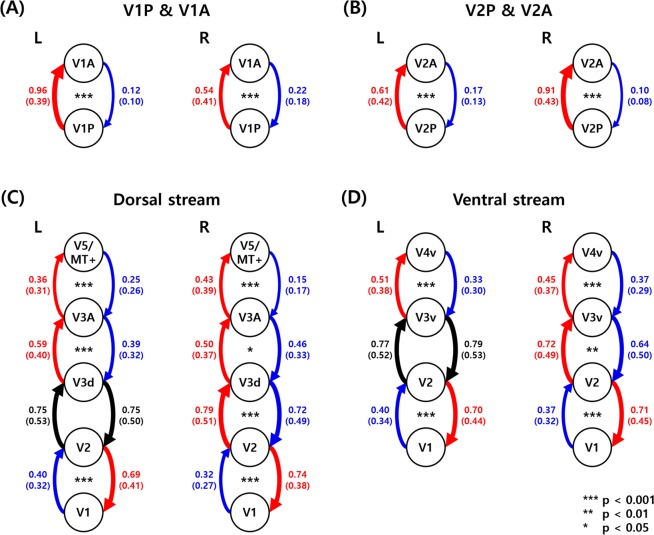
The mean strengths of the effective connectivity among the ROIs. Values are reported with a mean (SD) format. (**A**) The effective connectivity between V1A and V1P and (**B**) V2A and V2P. (**C**) The effective connectivity of the ROIs in the dorsal and (**D**) ventral streams. Between the two ROIs, the red line indicates the stronger strength of the effective connectivity while the blue line indicates the weaker connectivity strength. Black lines indicate the strengths of the effective connectivity that did not show significant differences between the two ROIs. The widths of the lines represent the strengths of the effective connectivity. If the thicker lines or red lines are shown on the left-hand side of the figures, subsequently they are consistent with the known signal flow in the visual streams. A, anterior; P, posterior; L, left hemisphere; R, right hemisphere.

To validate the reliability of the lag structure analysis, spectral DCM (spDCM) was performed to the same ROI sets. In the early visual cortices (i.e., V1 and V2), the strength of the effective connectivity from V1P to V1A was higher than that from V1A to V1P (Fig. 3A, *t* = 53.06 and *p* < 0.001 for the left hemisphere, *t* = 16.60 and *p* < 0.001 for the right hemisphere). The same patterns were identified between V2A and V2P (Fig. 3B, *t* = 23.37 and *p* < 0.001 for the left hemisphere, *t* = 46.76 and *p* < 0.001 for the right hemisphere).

The effective connectivity (obtained with spDCM) of the regions in the visual streams showed primarily consistent patterns with the results of the lag structure. In the dorsal stream, the strengths of the effective connectivity were consecutively organized except the connection between V1 and V2 (Fig. 3C, V1–V2: *t* = −14.44 and *p* < 0.001, V2 − V3d: *t* = −0.03 and *p* = 0.9787, V3d − V3A: *t* = 10.53 and *p* < 0.001, V3A − V5/MT+: *t* = 6.97 and *p* < 0.001 for the left hemisphere, V1 − V2: *t* = −23.26 and *p* < 0.001, V2 − V3d: *t* = 3.38 and *p* < 0.001, V3d − V3A: *t* = 2.54 and *p* = 0.0128, V3A − V5/MT+: *t* = 16.80 and *p* < 0.001 for the right hemisphere). The connectivity with positive *t*-values and small *p*-values could be the results from the flow of signal in the visual streams. Similar patterns were found in the ventral streams (Fig. 3D, V1 − V2: *t* = −13.62 and *p* < 0.001, V2 – V3v: *t* = −0.77 and *p* = 0.4660, V3v – V4v: *t* = 8.60 and *p* < 0.001 for the left hemisphere, V1 – V2: *t* = −15.51 and *p* < 0.001, V2 – V3v: *t* = 3.34 and *p* = 0.0011, V3v – V4v: *t* = 4.40 and *p* < 0.001 for the right hemisphere). The patterns of the effective connectivity were primarily consistent with those of the lag structure analysis, suggesting the visual streams might be the possible sources of the lag structure.

### Replication of the results with the independent dataset

The lag structure analysis was performed on the independent dataset of 595 participants with fast TR (0.645 s) obtained from the Enhanced Nathan Kline Institute-Rockland Sample (NKI-RS) database^28^ to validate our results. The order of temporal lag propagation paths between the anterior and posterior subregions of V1 and V2, and that of the dorsal and ventral streams are reported in Table S1 and Fig. S1. The total possible propagation paths between the subregions of V1 and V2 were 1,190 (=2 × 595). The temporal lag propagated from the posterior to the anterior subregions for bilateral V1 and V2 (Fig. S1A,B). Among 1,190 possible paths, 864 cases were observed for the left V1 (mean temporal lag = 0.2366 s), 846 cases for the right V1 (mean temporal lag = 0.2404 s), 778 cases for the left V2 (mean temporal lag = 0.2506 s), and 767 cases for the right V2 (mean temporal lag = 0.2656 s). The results of temporal lag propagation for visual streams are primarily consistent with our primary results. Among a total of 2,975 (=5 × 595) possible paths for the dorsal pathway, the path that was consistent with the dorsal stream was observed 135 times for the left hemisphere, and 137 times for the right hemisphere (the third and fourth highest frequencies, respectively) (Table S1 and Fig. S1C). For the ventral pathway, the path that was consistent with the ventral stream was observed 265 times for the left hemisphere, and 328 times for the right hemisphere (the second-highest frequency) among a total of 2,380 (=4 × 595) possible paths (Table S1 and Fig. S1D). The top three frequently observed propagation paths in the dorsal and ventral streams are plotted in Fig. S2. The results are primarily similar to the primary results derived from the HCP data. Further, spDCM was applied to the Enhanced NKI-RS data and exhibited consistent results as derived from the HCP data (Fig. S3). The results in the early visual cortices are as follows: V1A–V1P: *t* = 2.86 and *p* = 0.0133, V2A − V2P: *t* = 1.96 and *p* = 0.0762 for the left hemisphere, V1A − V1P: *t* = −7.17 and *p* < 0.001, V2A − V2P: *t* = 9.29 and *p* < 0.001 for the right hemisphere. The results in the dorsal stream are as follows: V1 − V2: *t* = 1.56 and *p* = 0.1534, V2 − V3d: *t* = 9.19 and *p* < 0.001, V3d − V3A: *t* = 6.43 and *p* < 0.001, V3A − V5/MT+: *t* = 0.79 and *p* = 0.4306 for the left hemisphere, V1 − V2: *t* = −2.25 and *p* = 0.0451, V2 − V3d: *t* = 13.52 and *p* < 0.001, V3d − V3A: *t* = 2.29 and *p* = 0.0451, V3A − V5/MT+: *t* = 2.00 and *p* = 0.0747 for the right hemisphere. The results in the ventral stream are as follows: V1 − V2: *t* = 1.34 and *p* = 0.2173, V2 − V3v: *t* = 1.58 and *p* = 0.1534, V3v − 4v: *t* = 2.26 and *p* = 0.0451 for the left hemisphere, V1 − V2: *t* = −1.09 and *p* = 0.2903, V2 − V3v: *t* = 2.25 and *p* = 0.0451, V3v − V4v: *t* = −1.17 and *p* = 0.2747 for the right hemisphere. We conclude that our results are well replicated with the independent dataset.

## Discussion

The existence of the temporal lag patterns in the fMRI signals was demonstrated in the previous studies^4,5,13^. Those studies suggested that the latency of fMRI signals could be attributed to the neural processes rather than the hemodynamic response delays^4,5^. However, the quantitative demonstration of the link between the neural processes and the lag structure is insufficient. In our study, we applied the lag structure analysis to the human visual cortex and found that the fMRI signal propagates from the posterior to the anterior subregions of V1 and V2, suggesting the central visual fields receive the information earlier than the peripheral visual fields consistent with the previous study^20^. In addition, we compared the lag structure results in the visual cortex with the dorsal and ventral streams. The temporal lag patterns were not exactly the same as the order of signal propagation in two visual streams, but they showed similar patterns. As an additional validation, we performed spDCM to estimate the strengths of the effective connectivity among the ROI sets, and the results from spDCM are similar to those from the lag structure. The results might imply that the neural signal flow in the visual streams might be the possible sources of the lag structure of fMRI signals in the human visual cortex. A previous review paper noted that linking the lag structure of the fMRI signals with neural activities was an important question^11^. Our results partly answered the question and reinforced the previous argument that the lag structure of fMRI signals reflects the neural processes at least for the visual cortex^4,5,11^. Our results might shed new insight into linking the temporal lag of fMRI signals and neural processes in the human brain.

Our lag structure analysis found that the propagation paths in the visual cortex were similar to the signal flows in visual streams. However, we also found several signal propagation paths that were different from the signal flows in visual streams. To see whether these propagation paths were related to the noise, we added white Gaussian noise to the original time series data and performed the lag structure analysis. Three levels of noise were considered at signal-to-noise ratio (SNR) of 8, 5, and 1 dB. The signal propagation path consistent with signal flows in visual streams stayed stable as the noise was added, but we observed new propagation paths inconsistent with the main results (Table 1) especially in the dorsal stream (Tables S2–S4). The results might indicate that the signal propagation paths inconsistent with the visual streams might be affected by the measurement noise.

In addition to the lag structure and spDCM analyses, we performed a zero-lag correlation analysis of the time series between V1 and other visual areas in two visual streams. This was to confirm whether a simple zero-lag correlation would be able to capture the lag structure related information. We found that the zero-lag correlation values showed a decreasing trend with respect to the order of signal flows in visual streams (Fig. S4). The decreasing trend was evident in the ventral stream, but such a trend was only observed for V5/MT+ in the dorsal stream. One possible explanation could be that the signal propagated along the visual streams and changed its shape, thus a simple zero-lag correlation might not fully capture the shape change.

We chose two visual streams for comparison with the lag structure of fMRI signals, as they are the well-studied representative neural pathways in the human visual cortex^14^. The dorsal stream is known as the “where” pathway that processes the location information of objects, and it propagates from the early visual cortices (V1 and V2) to the dorsal regions of the extrastriate visual cortices (V3d, V3A, and V5/MT+)^14,29–32^. The ventral stream is known as the “what” pathway that processes the information regarding object recognition and identification^14,29–32^. Unlike the dorsal stream, the ventral stream projects from the early visual cortices to the ventral regions of the extrastriate visual cortices (V3v and V4v)^14,29–32^. By investigating the relationship between the lag structure in the early visual cortices and the visual streams, we demonstrated the possible source of lag structure in human visual cortices. The source identification of temporal lag patterns in other neural pathways such as sensorimotor, auditory, and dopamine-related pathways is left for future studies.

To support and validate our results, we performed the same analyses to the two independent datasets from the HCP and NKI-RS databases. We found that the order of the signal propagation derived from the two databases was primarily consistent. Our results are well replicated with the independent dataset. The age distribution between the two databases was different. The participants in the HCP database were young adults (age range between 22 and 36), while those in the Enhanced NKI-RS database consisted of people from a wide age range (between 6 and 86). The lag structure analysis revealed robust results regardless of the age, suggesting the high reproducibility across a wide age span.

In this study, data from two independent databases were used. Participants of the HCP database kept their eyes open looking at a cross-hair on a screen during the scan^26^ while those of the Enhanced NKI-RS database kept their eyes open without any visual fixation^33^. Our results showed the signal propagation paths of the HCP data were more similar to the signal flows in two visual streams compared to those of the Enhanced NKI-RS data (Table 1 and S1, Figs 2, 3, S2 and S3) in terms of the observed frequency of the paths consistent with the visual streams. The results might suggest that the neural signal flows in the visual cortex became more active when a visual stimulus was given and that led to lag patterns of fMRI to be more consistent with the visual streams. Furthermore, the lag patterns might be altered by the degree of visual stimulus. Exploring the lag patterns using a richer visual stimulus such as watching a movie is an interesting topic which might provide further insights into possible sources of lag structure in visual cortex. This is left for future work.

Our study has a few limitations. First, it is known that the conventional temporal resolution of fMRI is relatively slow in capturing fast neural signals^34^. Previous studies explored the lag structure using fMRI with typical TR settings (approximately 2–3 s)^4,5,35^. To handle the issue of the temporal resolution, in this study, we used the fMRI data with TRs of 0.72 s and 0.645 s that are relatively faster than the typical TR. However, they are still slow to directly measure the neural activities; thus, future studies using fMRI data with a much faster TR is required for validation. Second, the temporal delays we observed were relatively slow and it could be due to measuring a venous blood flow that is slower than the neural process or a bona fide low-frequency phenomenon in the visual cortex. Distinguishing between the two possible explanations is an important question that needs further studies. Third, we resampled the fMRI data into 6 mm^3^ voxels for the efficient computation of a voxel-wise lag structure. The original dimensions of the fMRI data were 91 × 109 × 91 (=902,629 voxels), and the dimensionality-reduced data was of size 30 × 36 × 30 (=32,400 voxels). The 6 mm^3^ voxels was adopted in the previous studies, and it could be an appropriate voxel scale to compute the lag structure^4,5,13^. Fourth, we could not define V1 and V2 by retinotopy mapping owing to the limitation of the HCP and the Enhanced NKI-RS databases. Thus, we used the atlas of the anterior and posterior subregions of V1 and V2 defined using the HCP data in our previous study^27^. Our previous study showed that the anterior and posterior subregions of V1 and V2 were retinotopically mapped to the peripheral and central visual representations, respectively^27^, thus providing the rationale for using the predefined atlas. In future studies, we will collect large-scale retinotopic data to better define the visual areas.

In this study, we aimed to identify the possible sources of the lag structure in the human visual cortex using fMRI with fast TR settings. We found that the fMRI signal propagated from the posterior to the anterior subregion of early visual cortices suggesting the central visual fields process the brain information earlier than the peripheral visual fields. We also found that the patterns of the lag structure were similar with the dorsal and ventral pathways, suggesting that the visual streams might be the possible sources of the lag structure in the human visual cortex. The results of the lag structure were compared to those from effective connectivity computed with spDCM, and both results were similar. Our study suggested a possible link between the temporal lag of fMRI signals and neural processes in the human brain.

## Methods

### Subjects and imaging data

This retrospective study was approved by the Institutional Review Board (IRB) of Sungkyunkwan University, and it was performed in full accordance with the local IRB guidelines. Informed consent was obtained from all participants. The T1-weighted and T2-weighted structural MRI and rs-fMRI data of 1,206 participants were provided by the HCP database^26^. All MRI data were scanned using a Siemens 3T scanner housed at the Washington University. The image acquisition parameters of structural MRI data were as follows: number of slices = 256; voxel resolution = 0.7 mm^3^; flip angle = 8°; field of view (FOV) = 224 × 224 mm^2^; TR = 2,400 ms for T1-weighted and 3,200 ms for T2-weighted MRI data; echo time (TE) = 2.14 ms for T1-weighted and 565 ms for T2-weighted MRI data. The image acquisition parameters of the rs-fMRI data were as follows: number of slices = 72; voxel resolution = 2 mm^3^; flip angle = 52°; FOV = 208 × 108 mm^2^; TR = 720 ms; TE = 33.1 ms; number of volumes = 1,200. Participants were asked to maintain their eyes opened during the scan^26^. Participants with drug ingestion, color vision diseases, and family history of mental diseases were excluded. Participants without complete T1-weighted, T2-weighted, and rs-fMRI data were also excluded. Finally, 655 participants (56% females) were enrolled in this study. The mean age of the finally selected participants was 28.69 years with a standard deviation (SD) of 3.66 (ranging between 22 and 36).

An independent dataset of the T1-weighted and rs-fMRI data were obtained from the Enhanced NKI-RS database for validation^28^. The T1-weighted and rs-fMRI data were scanned using a 3T Siemens Magnetom Trio Tim scanner. The image acquisition parameters of the T1-weighted structural data were as follows: number of slices = 176; voxel resolution = 1 mm^3^; flip angle = 9°; FOV = 250 × 250 mm^2^; TR = 1,900 ms; TE = 2.52 ms. The image acquisition parameters of the rs-fMRI data were as follows: number of slices = 40; voxel resolution = 3 mm^3^; flip angle = 60°; FOV = 222 × 222 mm^2^; TR = 645 ms; TE = 30 ms; number of volumes = 900. Participants were asked to keep their eyes open during MRI scanning^33^. Participants without T1-weighted and rs-fMRI data were excluded from a total of 650 participants. Finally, 595 participants (61% female) of mean age 38.52 with SD 22.79 (ranging between 6 and 86) were enrolled in this study.

### Image preprocessing

The HCP data were already processed with minimal preprocessing steps using FSL and FreeSurfer software^36–38^. The structural MRI data were processed as follows: Gradient nonlinearity and b0 distortions were corrected. The T1-weighted and T2-weighted structural MRI data were registered onto the Montreal Neurological Institute (MNI) standard space. The skull was extracted by warping the MNI brain mask to the individual’s brain. The rs-fMRI data were processed as follows: Gradient distortions and head motions were corrected. The low-resolution fMRI data were registered onto the high-resolution structural MRI data, and subsequently onto the MNI standard space with 2 mm isotropic voxel resolution. The magnetic field inhomogeneity was corrected, and the skull was extracted by applying the MNI brain mask to the individual subject spaces. An intensity normalization of value 10,000 was applied to the four-dimensional (4D) fMRI data. The artificial components including head motion, cardiac and breathing cycles, and scanner artifacts were removed using FMRIB’s ICA-based X-noisefier (FIX) software^39^. The left-to-right and right-to-left phase-encoded rs-fMRI data were averaged^40^.

The Enhanced NKI-RS data were preprocessed using AFNI and FSL software^36,41^. The T1-weighted structural MRI data were processed as follows: The magnetic field inhomogeneity was corrected and the skull was removed. The rs-fMRI data were processed as follows: The volumes of the first 10 s were discarded to allow the magnetic field to be saturated. The volumes with large head movements (frame-wise displacement > 0.5 mm) were removed^42^. The slice timing and head motion were corrected and then intensity normalization of the all 4D volumes was applied with a value of 10,000. The fMRI data were registered to the T1-weighted structural MRI data and subsequently onto the MNI standard space. Nuisance variables including white matter, cerebrospinal fluid, head motion, and cardiac- and breathing-related contributions were removed using the FIX software^39^. A bandpass filter with 0.009–0.08 Hz and spatial smoothing with full-width at half-maximum of 6 mm were applied.

### Regions of interest

The ROIs were defined within the visual cortex (V1, V2, V3d, V3v, V3A, V4v, and V5/MT+) using the JuBrain atlas (Fig. S5)^43^. We divided the V1 and V2 atlases into the anterior (i.e., peripheral visual field) and posterior (i.e., central visual field) subregions using the connectivity-based parcellation approach to compare the lag structure between the peripheral and central visual fields^27^. Nine ROIs (V1A, V1P, V2A, V2P, V3d, V3v, V3A, V4v, V5/MT+) were used in this study (Fig. S5).

### Lag structure in early visual cortices

The conventional connectivity analysis was performed by calculating the Pearson correlation between the time series of different voxels^4,5^. Unlike the traditional approach, the lag structure analysis was computed by calculating the cross correlation between two different time series with variable temporal delays, and is represented in Equation (1)^4,5^.

1 $${C}_{{x}_{i}{x}_{j}}(\tau )=\frac{1}{T}\int {x}_{i}\,(t+\tau )\cdot {x}_{j}(t)\,dt$$

**Figure 4:**
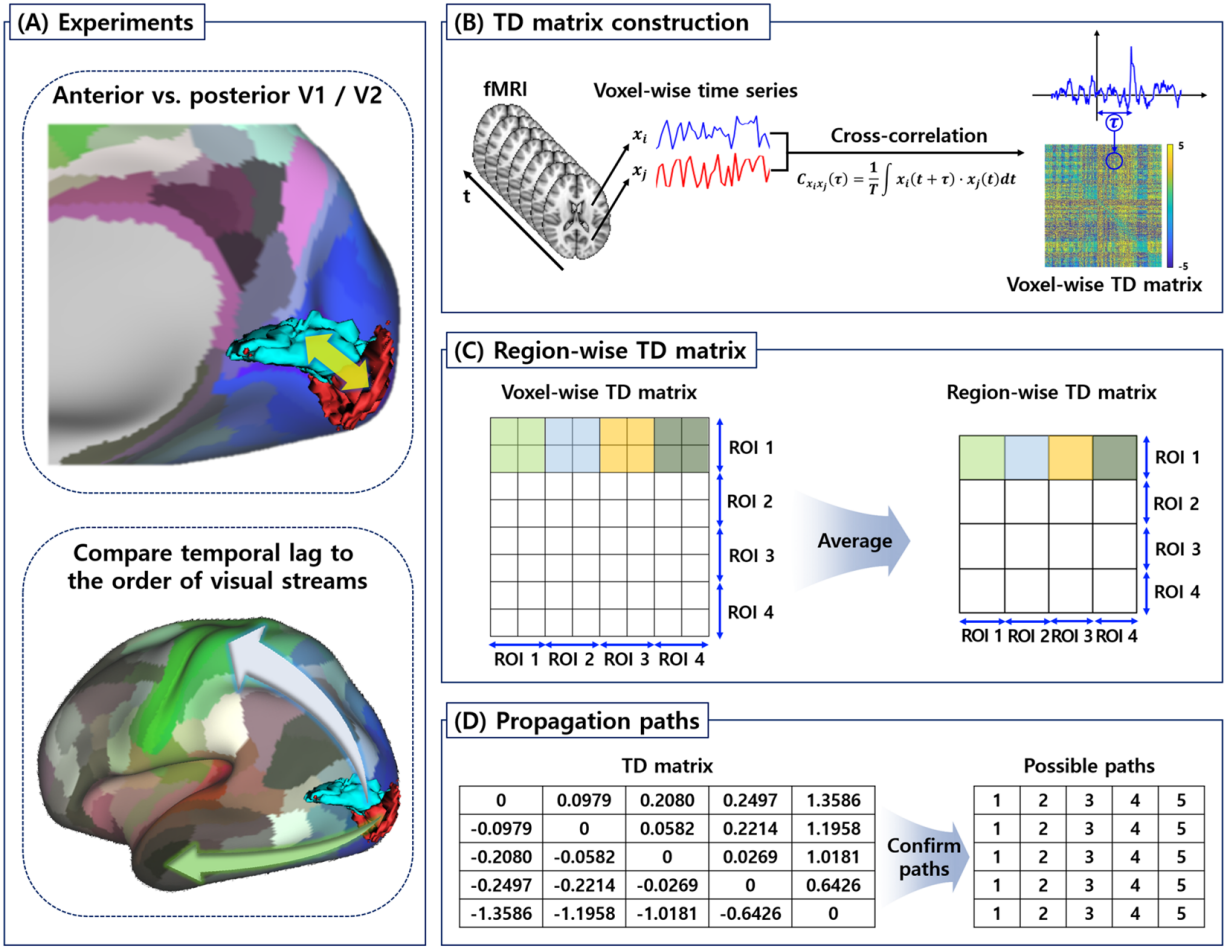
The overall flow of the study. (**A**) Two experimental settings. First, the temporal lag was compared between the anterior and posterior early visual cortices (V1 and V2). Second, the temporal lag was compared with the order of dorsal and ventral streams. (**B**) The procedure to construct the TD matrix. Voxel-wise time series were extracted from the rs-fMRI data and cross correlation was calculated to estimate the temporal lag *τ*. (**C**) The voxel-wise TD matrix was converted to the region-wise TD matrix by averaging the temporal lag within each ROI. (**D**) The possible propagation paths were generated from the region-wise TD matrix. TD, temporal delay; rs-fMRI, resting-state functional magnetic resonance imaging; ROI, region of interest.

${C}_{{x}_{i}{x}_{j}}$ is the cross-covariance function with respect to the temporal lag *τ* between the time series *x* of voxels *i* and *j*. To capture the sub-TR temporal delays, the time series was interpolated with the sampling frequency of 30 Hz. The temporal lag *τ* is determined, where ${C}_{{x}_{i}{x}_{j}}$ exhibits a positive or negative extremum value^4,5^. The cross correlation might reveal multiple extrema points; however, the time series of the fMRI signal is typically aperiodic, and thus it always yields a single extremum point^4,5^. To compute the lag structure between the central and peripheral visual fields (Fig. 4A), the cross correlation was computed throughout the voxels of the posterior and anterior subregions of V1 and V2. The temporal lag values were entered into a temporal delay (TD) matrix (Fig. 4B)^4,5^. The TD matrix is anti-symmetric, as the propagation from voxel *i* to *j* is the inverse of the propagation from voxel *j* to *i*. It is shown that the temporal delay of hemodynamic responses is typically 5 s to 6 s; thus, we set the TD matrix threshold as 5 s^44–47^. We transformed the voxel-wise TD matrix to the region-wise TD matrix by averaging the temporal lag values, as shown in the Fig. 4C. Temporal lags were initially computed on a voxel level, compared to region level, to perform measurements closer to a neuronal level. The rows of the region-wise TD matrix were sorted with respect to the temporal lag values, and several possible propagation paths were generated (Fig. 4D). The possible propagation paths were collected across all subjects and the most frequently observed path was considered as the significant path.

### Comparison with visual streams

To compare the lag structure in the visual cortex with the order of signal propagation in the dorsal and ventral streams (Fig. 4A), we defined two ROI sets. The first ROI set consisted of V1, V2, V3d, V3A, and V5/MT+ to represent the dorsal stream; the second ROI set consisted of V1, V2, V3v, and V4v to represent the ventral stream^48,49^. The lag structure was computed for the two ROI sets, and they were compared with the known order of signal propagation in the visual streams.

### Dynamic causal modeling

To compare the lag structure results with those from spDCM, we performed spDCM using statistical parametric mapping (SPM) 12. The spDCM was performed to the early visual cortices and the ROI sets in two visual streams^21–25^. The spDCM that estimates the neuronal dynamics and hemodynamic responses in the spectral domain was used as described in Equation (2) ^22^.

2 $$\begin{array}{rcl}\dot{x} & = & Ax+\nu \\ y & = & h\,(x,{\theta }_{h})+e,\,e \sim N(0,\Sigma ),\end{array}$$

where *x* is the hidden neuronal state for each ROI, *A* is the effective connectivity that represents the causal relationships between ROIs, and *v* is the endogenous neural fluctuations. The BOLD signal *y* is modeled using a nonlinear hemodynamic response function *h* that consisted of neuronal state *x* and parameters *θ*_*h*_^25^, and additive noise *e*. The spDCM was performed between the central and peripheral visual fields of early visual cortices (i.e., V1 and V2, respectively) and the ROI sets of the dorsal and ventral stream. The strengths of the effective connectivity were averaged across all subjects. The mean connectivity strength from region A to B was compared to that from region B to A using two-sample *t*-tests. The *p*-values were corrected using the false discovery rate.

## Supplementary information

Supplementary Information

## Data Availability

Part of the data is available from the HCP website (https://www.humanconnectome.org/). The HCP Institutional Data Access/Ethics Committee grants access to researchers who meet the criteria for access to the data. Another part of the data is available from the NKI website (http://fcon_1000.projects.nitrc.org/indi/enhanced/). We confirm that both HCP and NKI grants data access upon agreement with the data use policy.
