# Supplementary material for: Possible links between the lag structure in visual cortex and visual streams using fMRI
Source: Sci Rep. 2019 Mar 12;9:4283. doi: 10.1038/s41598-019-40728-x (PMC6414616; doi:10.1038/s41598-019-40728-x)
Supplement: Supplementary file 1 — Supplementary Information [file 41598_2019_40728_MOESM1_ESM.docx]

**Supplementary Information**

**Title: Possible links between the lag structure in visual cortex and visual streams using fMRI**

Authors: Bo-yong Park^1,2^, Won Mok Shim^2,3^, Oliver James^2^, and Hyunjin Park^2,4*^

^1^ Department of Electrical and Computer Engineering, Sungkyunkwan University, Suwon, 16419, Korea

^2^ Center for Neuroscience Imaging Research, Institute for Basic Science (IBS), Suwon, 16419, Korea

^3^ Department of Biomedical Engineering, Sungkyunkwan University, Suwon, 16419, Korea

^4^ School of Electronic and Electrical Engineering, Sungkyunkwan University, Suwon, 16419, Korea

*Corresponding Author:

Hyunjin Park, PhD

Center for Neuroscience Imaging Research

School of Electronic and Electrical Engineering

Sungkyunkwan University, Suwon, 16419, Korea

Phone: +82-31-299-4956

Fax: +82-31-290-5819

Email: [hyunjinp@skku.edu](mailto:hyunjinp@skku.edu)


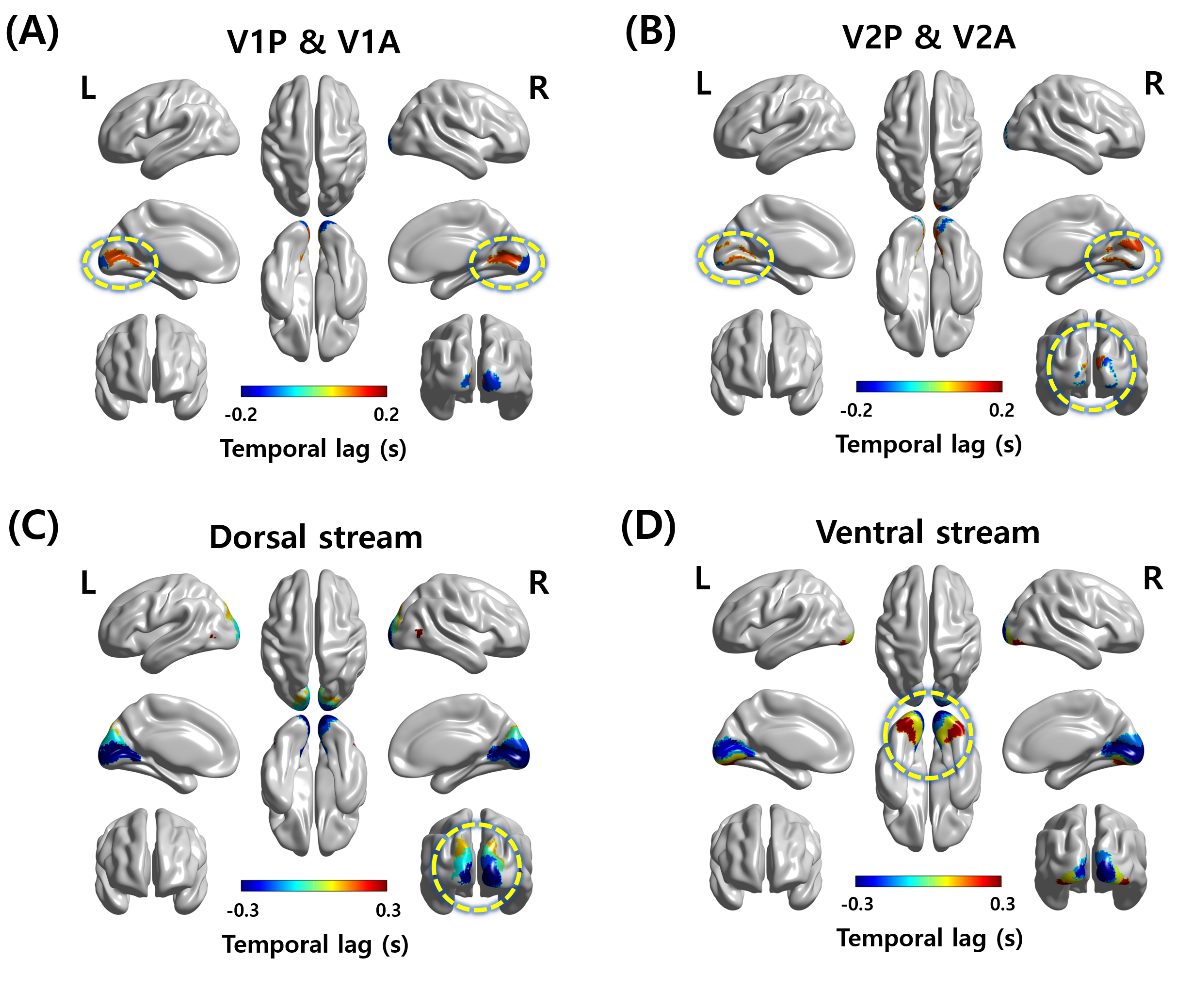


**Fig. S1.** The patterns of the temporal lag among ROIs using data from the Enhanced NKI-RS database. (A) The visualization of the temporal lag propagation path between V1A and V1P and (B) V2A and V2P. (C) The visualization of the temporal lag propagation path of the dorsal and (D) ventral streams. The most visible parts are marked with yellow dotted circles. A, anterior; P, posterior; L, left hemisphere; R, right hemisphere.


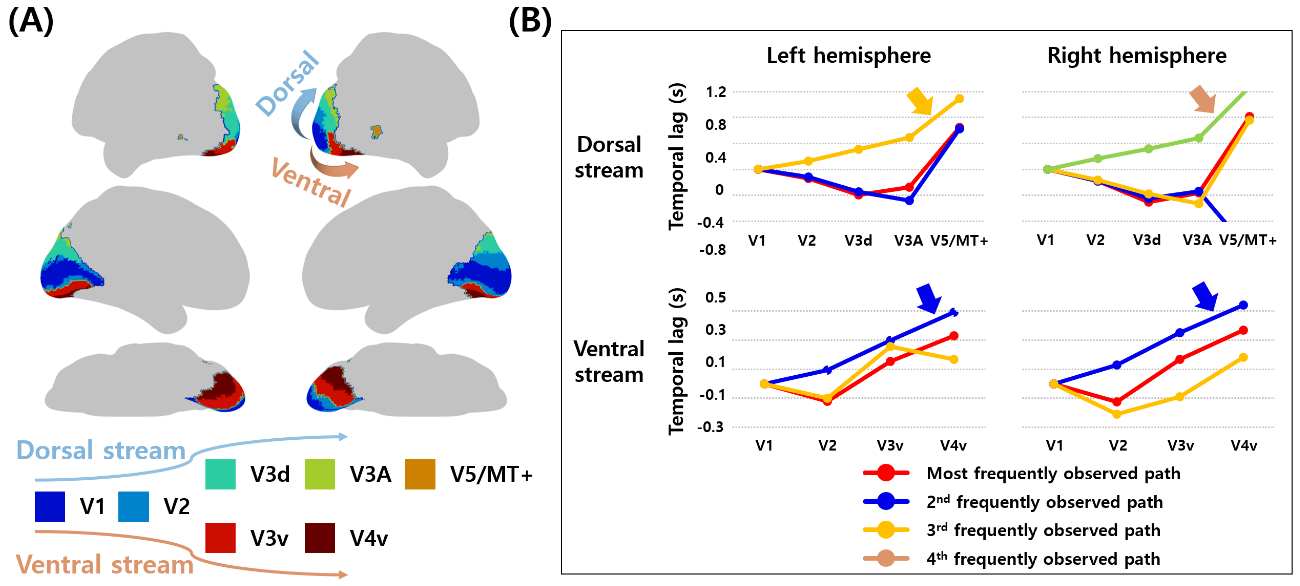


**Fig. S2.** The top three frequently observed propagation paths in dorsal and ventral streams using data from the Enhanced NKI-RS database. (A) The ROIs of dorsal and ventral streams. (B) The top three frequently observed paths according to the temporal lag values are plotted with different colors. The temporal lag value in V1 was set to zero and those for other regions were moderated. The paths that were consistent with the order of the visual streams are reported with arrows.


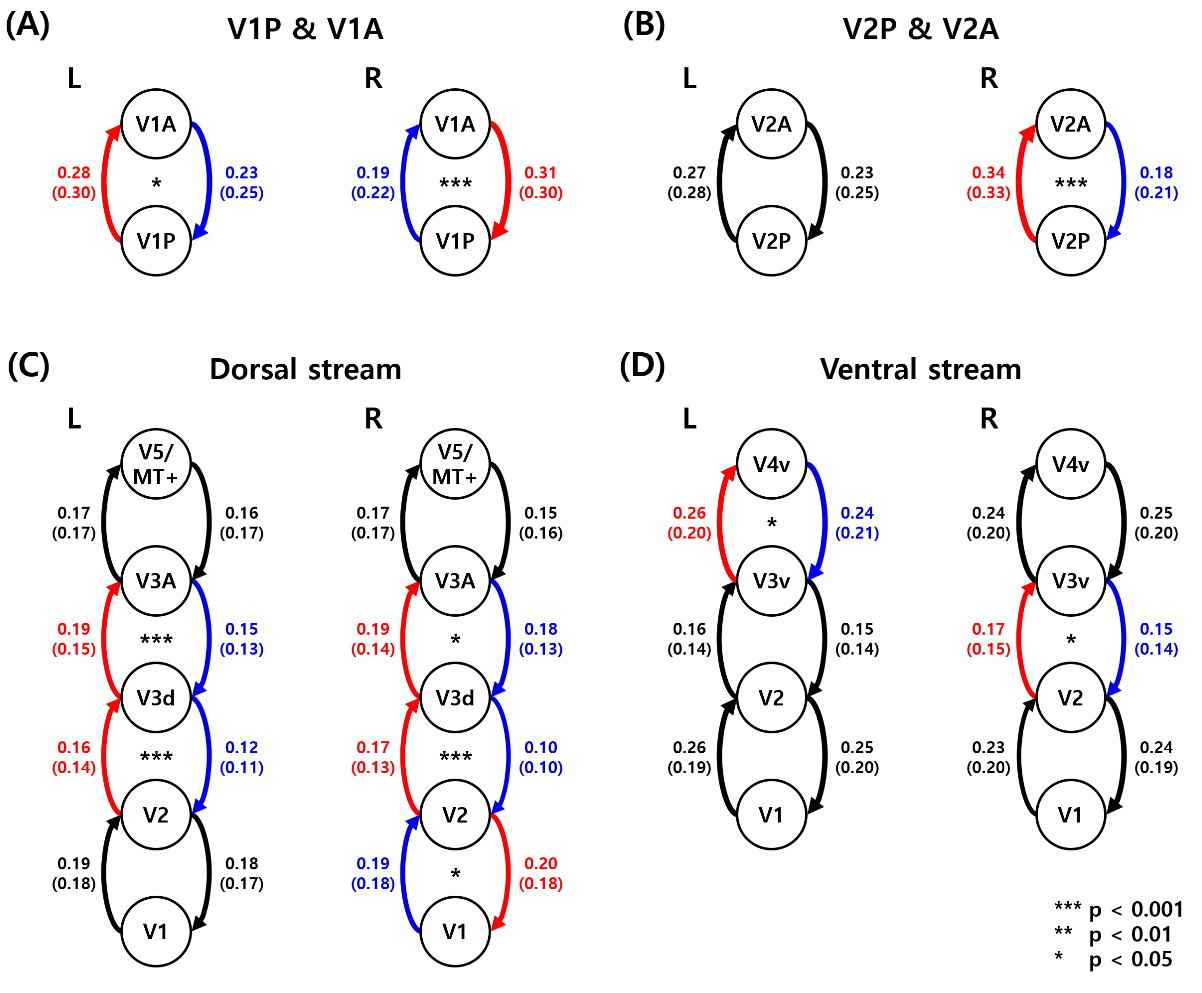


**Fig. S3.** The mean strengths of the effective connectivity among the ROIs using data from the Enhanced NKI-RS database. Values are reported with a mean (SD) format. (A) The effective connectivity between V1A and V1P and (B) V2A and V2P. (C) The effective connectivity of the ROIs in the dorsal and (D) ventral streams. Between the two ROIs, the red line indicates the stronger strength of the effective connectivity while the blue line indicates weaker connectivity strength. Black lines indicate the strengths of the effective connectivity that did not show significant differences between the two ROIs. The widths of the lines represent the strengths of the effective connectivity. If the thicker lines or red lines are shown on the left-hand side of the figures, subsequently they are consistent with the known signal flow in visual streams. A, anterior; P, posterior; L, left hemisphere; R, right hemisphere.


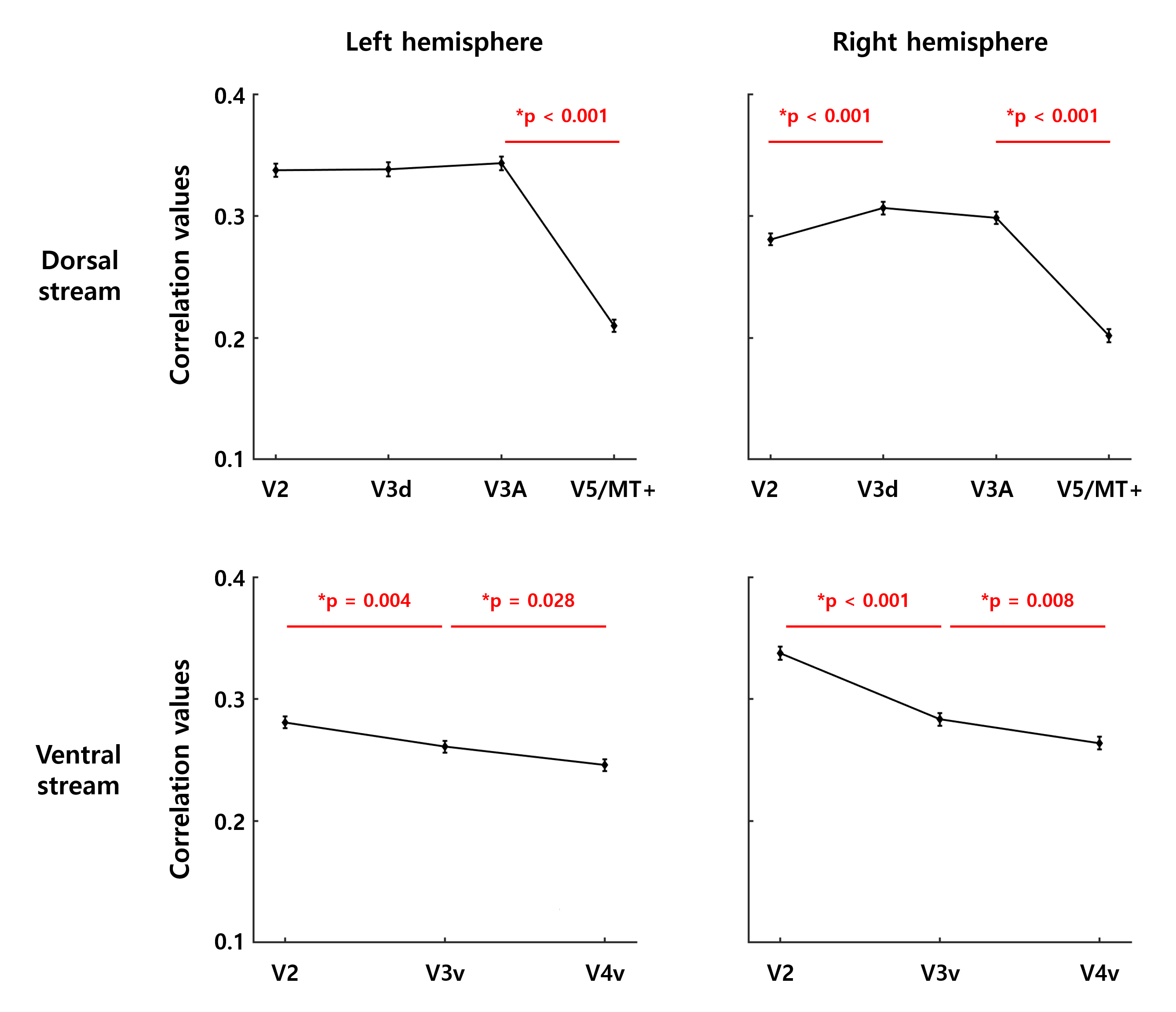


**Fig. S4.** The correlation (i.e., zero-lag) values between V1 and other visual areas in dorsal and ventral streams, respectively. Error bars represent ±1 standard error of the mean. The *p*-values were corrected with the false discovery rate.


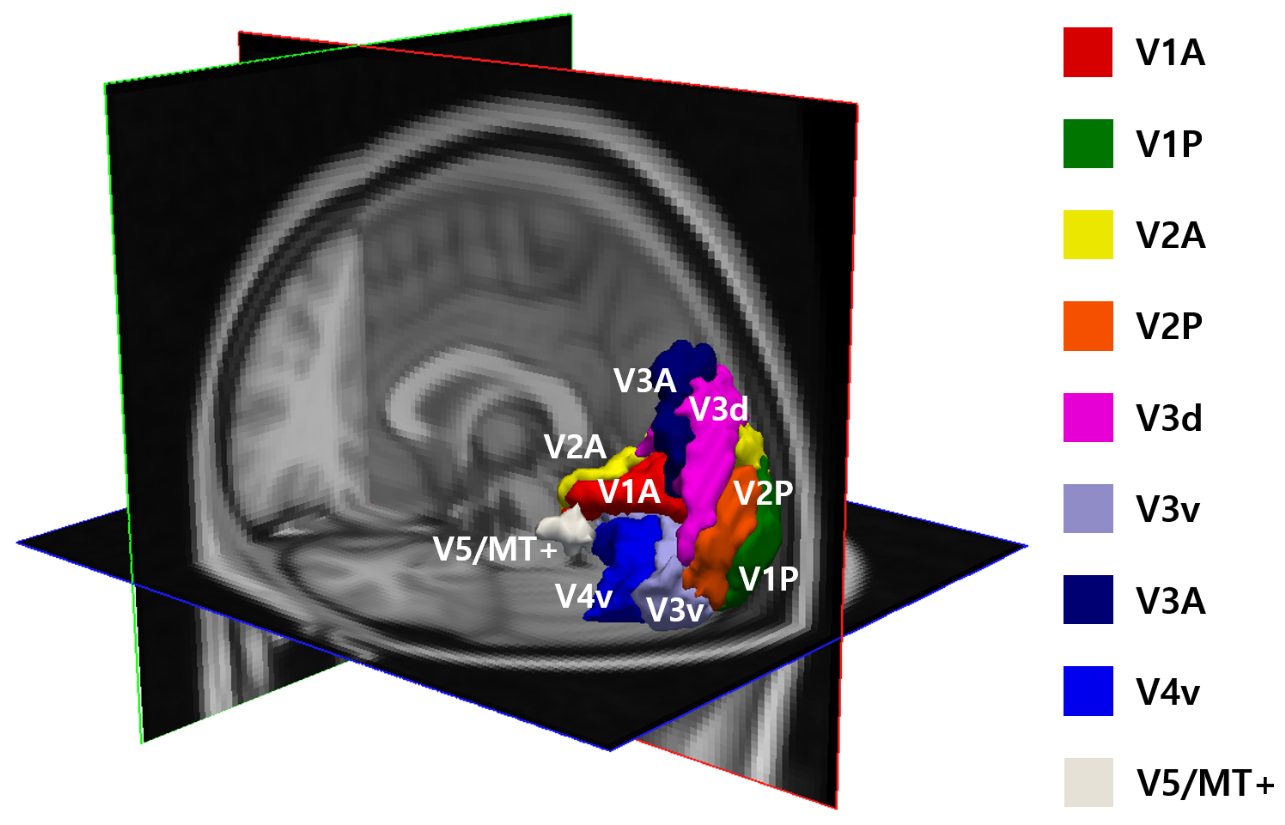


**Fig. S5.** The ROIs used in this study. Each ROI was reported with different colors. A, anterior; P, posterior; MT+, middle temporal.

**Table S1.** The top three frequently observed propagation paths in dorsal and ventral streams using data from the Enhanced NKI-RS database. The regions and corresponding mean temporal lag values (unit in seconds) were reported. The paths that showed the same propagation order with visual streams were reported in bold italic.

| **Stream** | **Frequency** | **Propagation paths and temporal lag (s)** | | | | | | | |
| --- | --- | --- | --- | --- | --- | --- | --- | --- | --- |
| Left dorsal | 1^st^ (184 times) | V3d | V3A | | V2 | | V1 | | V5/MT+ |
|  |  | -0.2601 | -0.1378 | | -0.0047 | | 0.1336 | | 0.7844 |
|  | 2^nd^ (146 times) | V3A | V3d | | V2 | | V1 | | V5/MT+ |
|  |  | -0.3495 | -0.2163 | | 0.0114 | | 0.1297 | | 0.7579 |
|  | ***3^rd^ (135 times)*** | ***V1*** | ***V2*** | | ***V3d*** | | ***V3A*** | | ***V5/MT+*** |
|  |  | ***-0.3886*** | ***-0.2590*** | | ***-0.0753*** | | ***0.1055*** | | ***0.7063*** |
| Right dorsal | 1^st^ (193 times) | V3d | V3A | | V2 | | V1 | | V5/MT+ |
|  |  | -0.3405 | -0.1973 | | -0.0095 | | 0.1668 | | 0.9852 |
|  | 2^nd^ (146 times) | V5/MT | V3d | | V3A | | V2 | | V1 |
|  |  | -0.9666 | -0.1552 | | -0.0403 | | 0.1174 | | 0.2963 |
|  | 3^rd^ (141 times) | V3A | V3d | | V2 | | V1 | | V5/MT+ |
|  |  | -0.3899 | -0.2356 | | -0.0265 | | 0.1381 | | 0.9000 |
|  | ***4^th^ (137 times)*** | ***V1*** | ***V2*** | | ***V3d*** | | ***V3A*** | | ***V5/MT+*** |
|  |  | ***-0.3532*** | ***-0.1877*** | | ***-0.0350*** | | ***0.1323*** | | ***0.9162*** |
| Left ventral | 1^st^ (392 times) | V2 | | V1 | | V3v | | V4v | |
|  |  | -0.2072 | | -0.0876 | | 0.0641 | | 0.2405 | |
|  | ***2^nd^ (265 times)*** | ***V1*** | | ***V2*** | | ***V3v*** | | ***V4v*** | |
|  |  | ***-0.2243*** | | ***-0.1312*** | | ***0.0737*** | | ***0.2669*** | |
|  | 3^rd^ (190 times) | V2 | | V1 | | V4v | | V3v | |
|  |  | -0.1866 | | -0.0863 | | 0.0805 | | 0.1701 | |
| Right ventral | 1^st^ (404 times) | V2 | | V1 | | V3v | | V4v | |
|  |  | -0.2118 | | -0.0864 | | 0.0818 | | 0.2813 | |
|  | ***2^nd^ (328 times)*** | ***V1*** | | ***V2*** | | ***V3v*** | | ***V4v*** | |
|  |  | ***-0.2806*** | | ***-0.1528*** | | ***0.0684*** | | ***0.2585*** | |
|  | 3^rd^ (185 times) | V2 | | V3v | | V1 | | V4v | |
|  |  | -0.1874 | | -0.0674 | | 0.0232 | | 0.2032 | |

**Table S2.** The top three frequency observed propagation paths in dorsal and ventral streams when the white Gaussian noise of 8 dB was added. The regions and corresponding mean temporal lag values (unit in seconds) were reported. The paths that showed the same propagation order with visual streams were reported in bold italic.

| **Stream** | **Frequency** | **Propagation paths and temporal lag (s)** | | | | | | | |
| --- | --- | --- | --- | --- | --- | --- | --- | --- | --- |
| Left dorsal | ***1^st^ (322 times)*** | ***V1*** | ***V2*** | | ***V3d*** | | ***V3A*** | | ***V5/MT+*** |
|  |  | ***-0.3518*** | ***-0.2139*** | | ***-0.0728*** | | ***0.0795*** | | ***0.5198*** |
|  | 2^nd^ (261 times) | V2 | V1 | | V3d | | V3A | | V5/MT+ |
|  |  | -0.2880 | -0.1995 | | -0.0560 | | 0.1210 | | 0.5239 |
|  | 3^rd^ (186 times) | V3d | V2 | | V1 | | V3A | | V5/MT+ |
|  |  | -0.3078 | -0.1871 | | -0.0804 | | 0.0540 | | 0.5072 |
| Right dorsal | ***1^st^ (233 times)*** | ***V1*** | ***V2*** | | ***V3d*** | | ***V3A*** | | ***V5/MT+*** |
|  |  | ***-0.3655*** | ***-0.2093*** | | ***-0.0779*** | | ***0.0573*** | | ***0.5812*** |
|  | 1^st^ (233 times) | V3d | V3A | | V2 | | V1 | | V5/MT+ |
|  |  | -0.3517 | -0.2160 | | -0.0602 | | 0.1392 | | 0.7624 |
|  | 3^rd^ (203 times) | V3d | V2 | | V3A | | V1 | | V5/MT+ |
|  |  | -0.2952 | -0.1707 | | -0.0661 | | 0.0816 | | 0.6585 |
| Left ventral | 1^st^ (398 times) | V2 | | V1 | | V3v | | V4v | |
|  |  | -0.1941 | | -0.0829 | | 0.0788 | | 0.2342 | |
|  | ***2^nd^ (308 times)*** | ***V1*** | | ***V2*** | | ***V3v*** | | ***V4v*** | |
|  |  | ***-0.1979*** | | ***-0.1129*** | | ***0.0619*** | | ***0.2105*** | |
|  | 3^rd^ (203 times) | V1 | | V2 | | V4v | | V3v | |
|  |  | -0.1869 | | -0.0894 | | 0.0792 | | 0.1972 | |
| Right ventral | 1^st^ (506 times) | V2 | | V1 | | V3v | | V4v | |
|  |  | -0.2142 | | -0.0831 | | 0.0416 | | 0.2675 | |
|  | 2^nd^ (337 times) | V2 | | V3v | | V1 | | V4v | |
|  |  | -0.2044 | | -0.0791 | | 0.0199 | | 0.2156 | |
|  | ***3^rd^ (262 times)*** | ***V1*** | | ***V2*** | | ***V3v*** | | ***V4v*** | |
|  |  | ***-0.2164*** | | ***-0.1240*** | | ***0.0417*** | | ***0.2283*** | |

**Table S3.** The top three frequency observed propagation paths in dorsal and ventral streams when the white Gaussian noise of 5 dB was added. The regions and corresponding mean temporal lag values (unit in seconds) were reported. The paths that showed the same propagation order with visual streams were reported in bold italic.

| **Stream** | **Frequency** | **Propagation paths and temporal lag (s)** | | | | | | | |
| --- | --- | --- | --- | --- | --- | --- | --- | --- | --- |
| Left dorsal | ***1^st^ (309 times)*** | ***V1*** | ***V2*** | | ***V3d*** | | ***V3A*** | | ***V5/MT+*** |
|  |  | ***-0.3393*** | ***-0.2175*** | | ***-0.0587*** | | ***0.0965*** | | ***0.5701*** |
|  | 2^nd^ (264 times) | V2 | V1 | | V3d | | V3A | | V5/MT+ |
|  |  | -0.2801 | -0.1827 | | -0.0625 | | 0.1222 | | 0.5211 |
|  | 3^rd^ (185 times) | V3d | V2 | | V1 | | V3A | | V5/MT+ |
|  |  | -0.2837 | -0.1586 | | -0.0492 | | 0.0782 | | 0.5747 |
| Right dorsal | 1^st^ (257 times) | V3d | V3A | | V2 | | V1 | | V5/MT+ |
|  |  | -0.3478 | -0.2179 | | -0.0469 | | 0.1449 | | 0.7331 |
|  | ***2^nd^ (223 times)*** | ***V1*** | ***V2*** | | ***V3d*** | | ***V3A*** | | ***V5/MT+*** |
|  |  | ***-0.3685*** | ***-0.2044*** | | ***-0.0682*** | | ***0.0738*** | | ***0.5922*** |
|  | 3^rd^ (206 times) | V3d | V2 | | V3A | | V1 | | V5/MT+ |
|  |  | -0.2935 | -0.1592 | | -0.0543 | | 0.0935 | | 0.7422 |
| Left ventral | 1^st^ (386 times) | V2 | | V1 | | V3v | | V4v | |
|  |  | -0.1938 | | -0.0824 | | 0.0797 | | 0.2366 | |
|  | ***2^nd^ (320 times)*** | ***V1*** | | ***V2*** | | ***V3v*** | | ***V4v*** | |
|  |  | ***-0.2011*** | | ***-0.1182*** | | ***0.0617*** | | ***0.2164*** | |
|  | 3^rd^ (244 times) | V1 | | V2 | | V4v | | V3v | |
|  |  | -0.1921 | | -0.0934 | | 0.0878 | | 0.2003 | |
| Right ventral | 1^st^ (500 times) | V2 | | V1 | | V3v | | V4v | |
|  |  | -0.2312 | | -0.0934 | | 0.0383 | | 0.2695 | |
|  | 2^nd^ (346 times) | V2 | | V3v | | V1 | | V4v | |
|  |  | -0.1867 | | -0.0684 | | 0.0318 | | 0.2315 | |
|  | ***3^rd^ (274 times)*** | ***V1*** | | ***V2*** | | ***V3v*** | | ***V4v*** | |
|  |  | ***-0.2085*** | | ***-0.1248*** | | ***0.0484*** | | ***0.2327*** | |

**Table S4.** The top three frequency observed propagation paths in dorsal and ventral streams when the white Gaussian noise of 1 dB was added. The regions and corresponding mean temporal lag values (unit in seconds) were reported. The paths that showed the same propagation order with visual streams were reported in bold italic.

| **Stream** | **Frequency** | **Propagation paths and temporal lag (s)** | | | | | | | |
| --- | --- | --- | --- | --- | --- | --- | --- | --- | --- |
| Left dorsal | ***1^st^ (331 times)*** | ***V1*** | ***V2*** | | ***V3d*** | | ***V3A*** | | ***V5/MT+*** |
|  |  | ***-0.3616*** | ***-0.2274*** | | ***-0.0782*** | | ***0.0909*** | | ***0.6103*** |
|  | 2^nd^ (269 times) | V2 | V1 | | V3d | | V3A | | V5/MT+ |
|  |  | -0.2635 | -0.1697 | | -0.0451 | | 0.1555 | | 0.6053 |
|  | 3^rd^ (188 times) | V3d | V3A | | V2 | | V1 | | V5/MT+ |
|  |  | -0.3406 | -0.1778 | | -0.0307 | | 0.1119 | | 0.5360 |
| Right dorsal | 1^st^ (296 times) | V3d | V3A | | V2 | | V1 | | V5/MT+ |
|  |  | -0.3816 | -0.2362 | | -0.0714 | | 0.1249 | | 0.7711 |
|  | 2^nd^ (220 times) | V3d | V2 | | V3A | | V1 | | V5/MT+ |
|  |  | -0.3103 | -0.1694 | | -0.0698 | | 0.0692 | | 0.7815 |
|  | ***3^rd^ (209 times)*** | ***V1*** | ***V2*** | | ***V3d*** | | ***V3A*** | | ***V5/MT+*** |
|  |  | ***-0.3306*** | ***-0.1787*** | | ***-0.0430*** | | ***0.1140*** | | ***0.6835*** |
| Left ventral | 1^st^ (423 times) | V2 | | V1 | | V3v | | V4v | |
|  |  | -0.2043 | | -0.0921 | | 0.0814 | | 0.2440 | |
|  | ***2^nd^ (343 times)*** | ***V1*** | | ***V2*** | | ***V3v*** | | ***V4v*** | |
|  |  | ***-0.1835*** | | ***-0.1066*** | | ***0.0765*** | | ***0.2370*** | |
|  | 3^rd^ (245 times) | V1 | | V2 | | V4v | | V3v | |
|  |  | -0.2085 | | -0.1055 | | 0.0809 | | 0.1933 | |
| Right ventral | 1^st^ (530 times) | V2 | | V1 | | V3v | | V4v | |
|  |  | -0.2317 | | -0.0912 | | 0.0418 | | 0.2802 | |
|  | 2^nd^ (334 times) | V2 | | V3v | | V1 | | V4v | |
|  |  | -0.1918 | | -0.0691 | | 0.0318 | | 0.2374 | |
|  | ***3^rd^ (305 times)*** | ***V1*** | | ***V2*** | | ***V3v*** | | ***V4v*** | |
|  |  | ***-0.2206*** | | ***-0.1287*** | | ***0.0538*** | | ***0.2590*** | |
